# Supplementary material for: Hybridisation has shaped a recent radiation of grass-feeding aphids
Source: BMC Biol. 2023 Jul 13;21:157. doi: 10.1186/s12915-023-01649-4 (PMC10347838; doi:10.1186/s12915-023-01649-4)
Supplement: Supplementary file 2 — Additional file 2: Figures S1 - S36. Figure S1. Whole genome alignment of the S. miscanthi v1 and A. pisum JIC1 v1 genome assemblies. Figure S2. BUSCO completeness plot. Figures S3, S5 and S8. KAT k-mer spectra plots. Figures S4, S6, S9, S25 - S30, S33 and S44. Taxon-annotated GC content-coverage plots. Figures S7, S24, S31 and S32. Hi-C contact maps. Figure S10. Gene set BUSCO completeness plot. Figure S11. Maximum likelihood phylogeny of 11 aphid species. Figure S12. JIC1 and LF1 within individual haplotype divergence. Figure S13. JIC1 and LF1 between individual haplotype divergence. Figures S14 - S21. Per chromosome SplitsTree networks of phased haplotypes for S. miscanthi and S. avaenae GBS samples and the Langfang-1 and JIC1 whole genome samples. Figure S22. Synonymous site divergence between S. miscanthi and M. dirhodum one-to-one orthologs. Figure S23. SNAPP maximum-clade-credibility time calibrated phylogeny of Sitobion lineages. Figures S35 and S36. Per haplotype, per chromosome sequencing depth for the Hapcut2 phased assembly of S. miscanthi Langfang-1 based on Illuminaand PacBioread mapping. [file 12915_2023_1649_MOESM2_ESM.docx]

**Supplementary Figures**


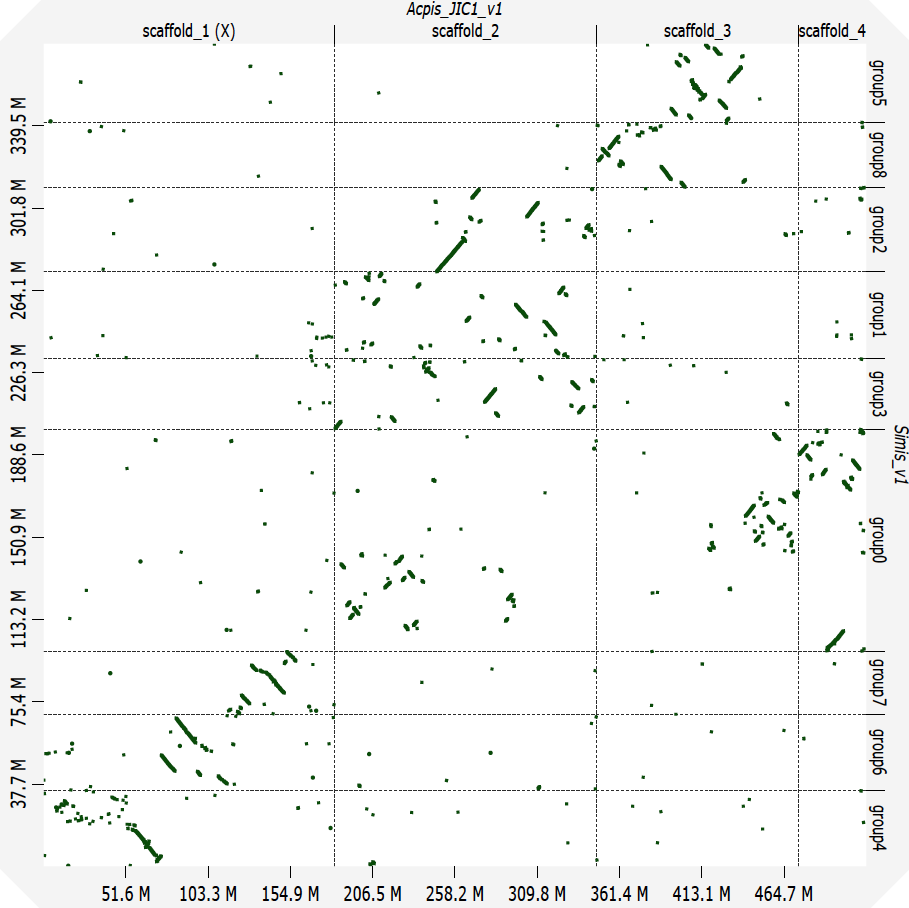


**Figure S1:** *Dot plot* showing whole genome alignment of the *Sitobion miscanthi* v1 (y-axis; Simis_v1) and *Acyrthosiphon pisum* JIC1 v1 (x-axis; Acpis_JIC1_v1) genome assemblies. Simis_v1 scaffolds are ordered along the Acpis_JIC1_v1 assembly. For clarity, only chromosome scale genomic scaffolds are aligned. The x- and y-axis show cumulative scaffold length in Mb.


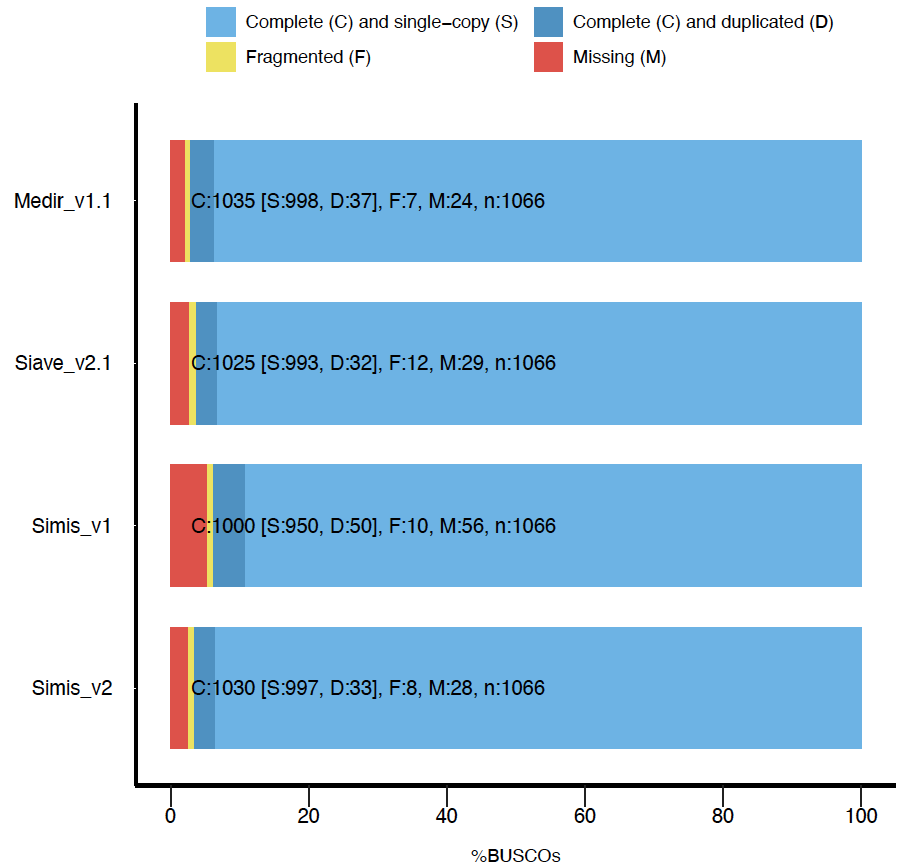


**Figure S2:** BUSCO completeness plot for *Metopolophium dirhodum* v1.1 (Medir_v1.1), *Sitobion avenae* JIC1 v2.1 (Siave_v2.1), *Sitobion miscanthi* v1 (Simis_v1) and *Sitobion miscanthi* v2 (Simis_v2) genome assemblies. The genomes were assessed using BUSCO v3 and the Arthropoda gene set (odb9; n=1,066).


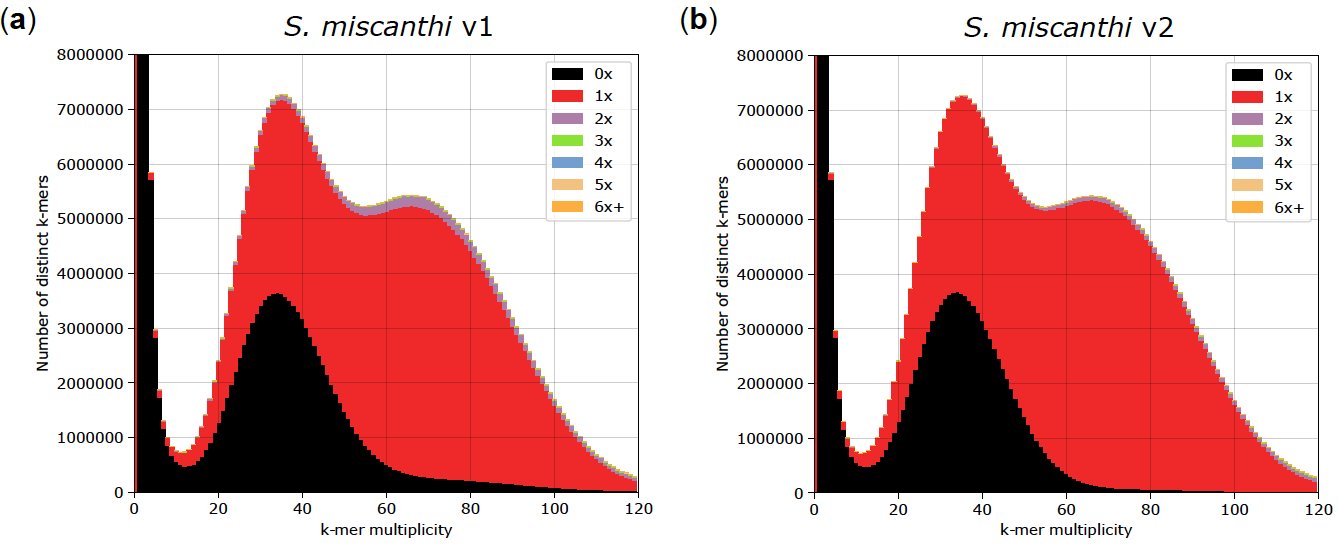


**Figure S3:** KAT k-mer spectra plot comparing *S. miscanthi* Illumina paired-end (PE) reads from Jiang *et al.* (2019) to the original *S. miscanthi* assembly (**a**) and our updated *S. miscanthi* assembly (**b**). Colours indicate how many times fixed length words (k-mers) from the reads appear in the assembly. Red indicates k-mers found only once in the assembly, black indicates content present in the reads but missing from the assembly and other colours indicate k-mers that are duplicated in the assembly. The x-axis shows the number of times each k-mer is found in the reads (k-mer multiplicity) and the y-axis shows the count of distinct k-mers in 1x k-mer multiplicity bins.


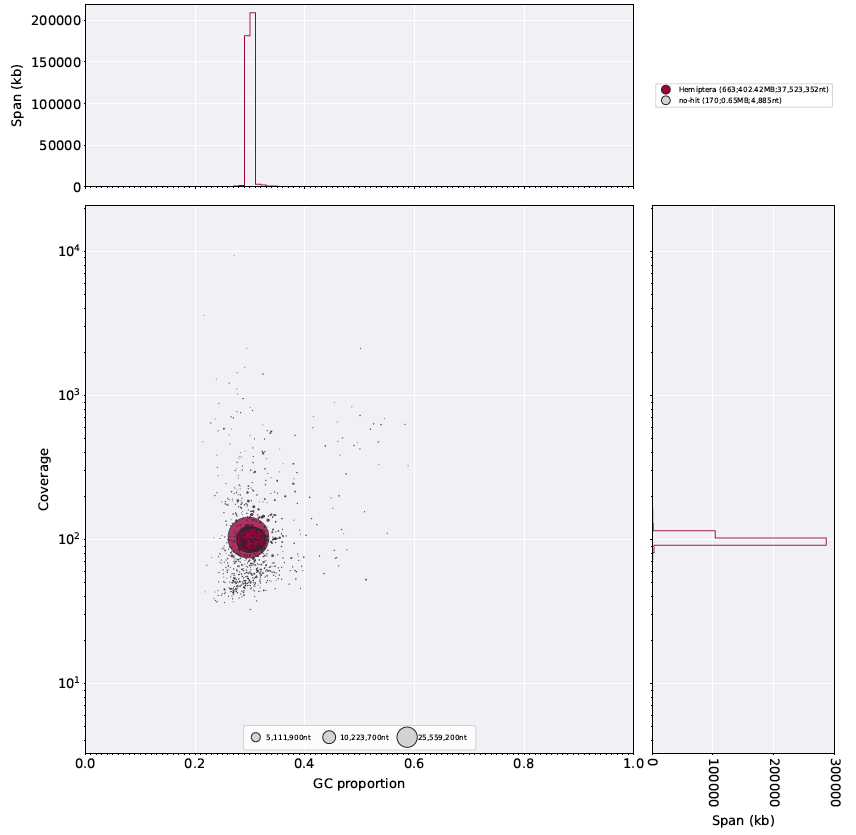


**Figure S4:** Taxon-annotated GC content-coverage plot of the *Sitobion miscanthi* v2 genome assembly. Each circle represents a scaffold in the assembly, scaled by length, and coloured by order-level NCBI taxonomy assigned by BlobTools. The x-axis corresponds to the average GC content of each scaffold and the y-axis corresponds to the average coverage based on alignment with Illumina paired-end reads from the *S. miscanthi* Langfang-1 colony (from Jiang *et al.* 2019). Marginal histograms show cumulative genome content (in Kb) for bins of coverage (y-axis) and GC content (x-axis).


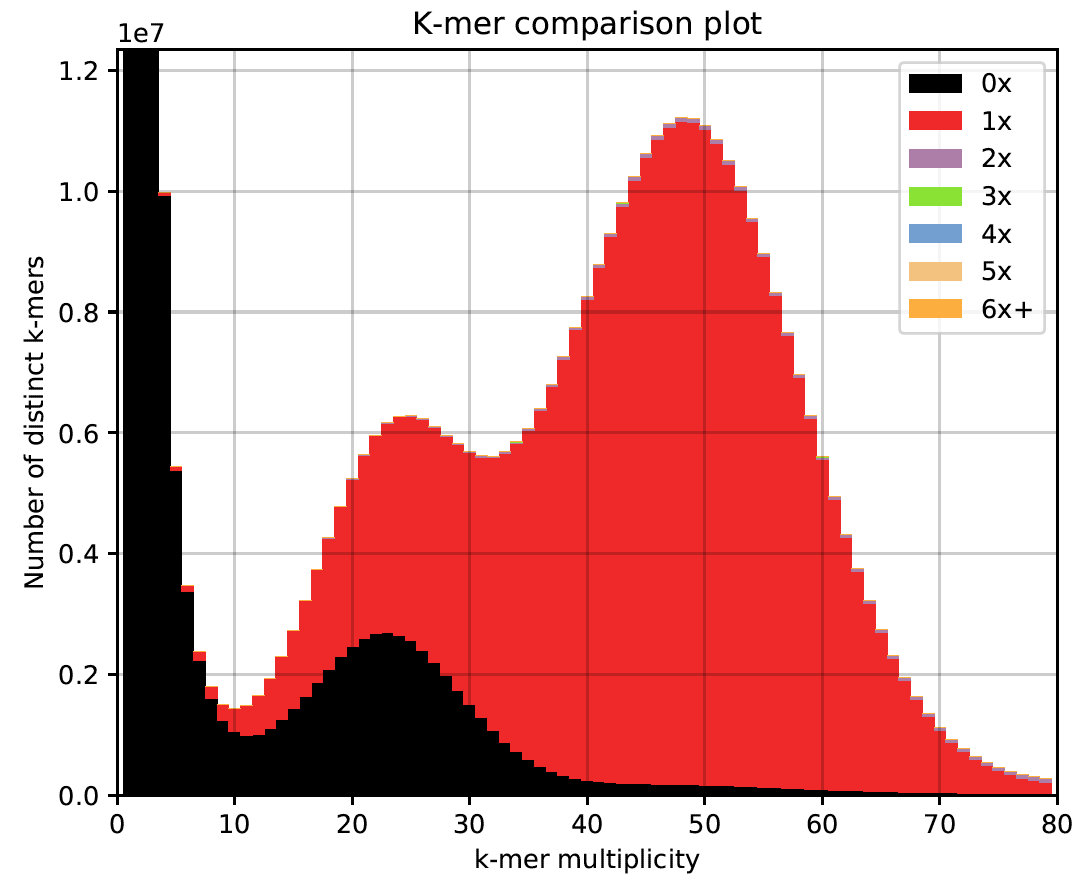


**Figure S5:** KAT k-mer spectra plot comparing genomic *S. avenae* Illumina paired-end (PE) reads to the *S. avenae* v2.1 genome assembly. See **Figure S3** legend for detailed description of the plot.


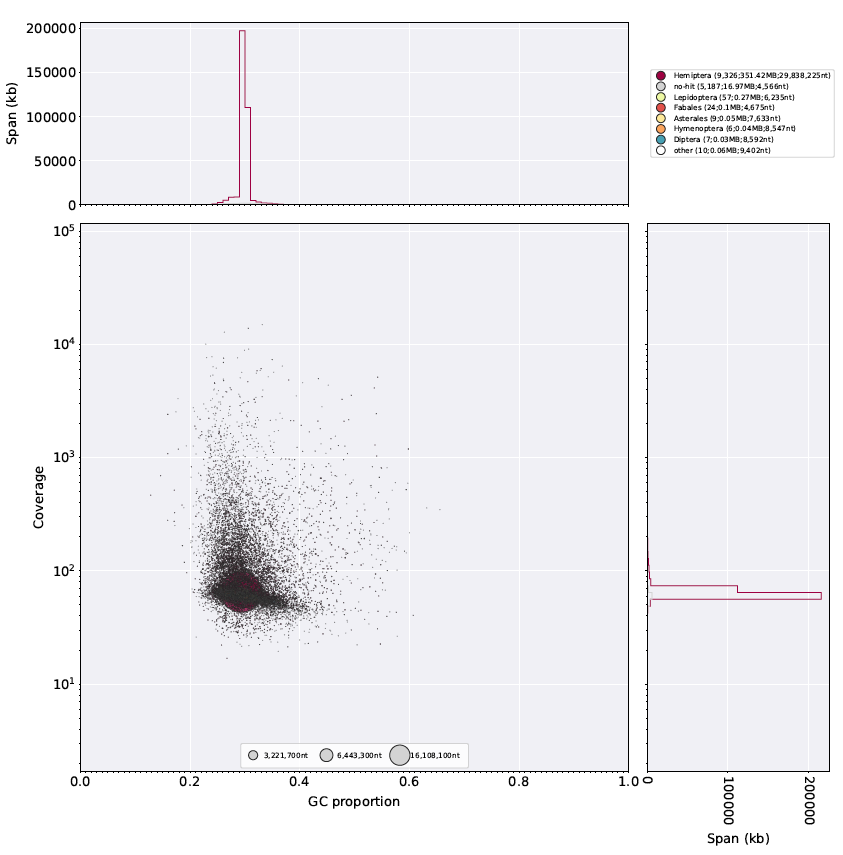


**Figure S6:** Taxon-annotated GC content-coverage plot of the *Sitobion avenae* v2.1 genome assembly. Scaffold coverage (y-axis) is based on alignment of PCR-free Illumina paired-end reads from the *S. avenae* JIC insectary colony. See **Figure S4** legend for detailed description of the plot.


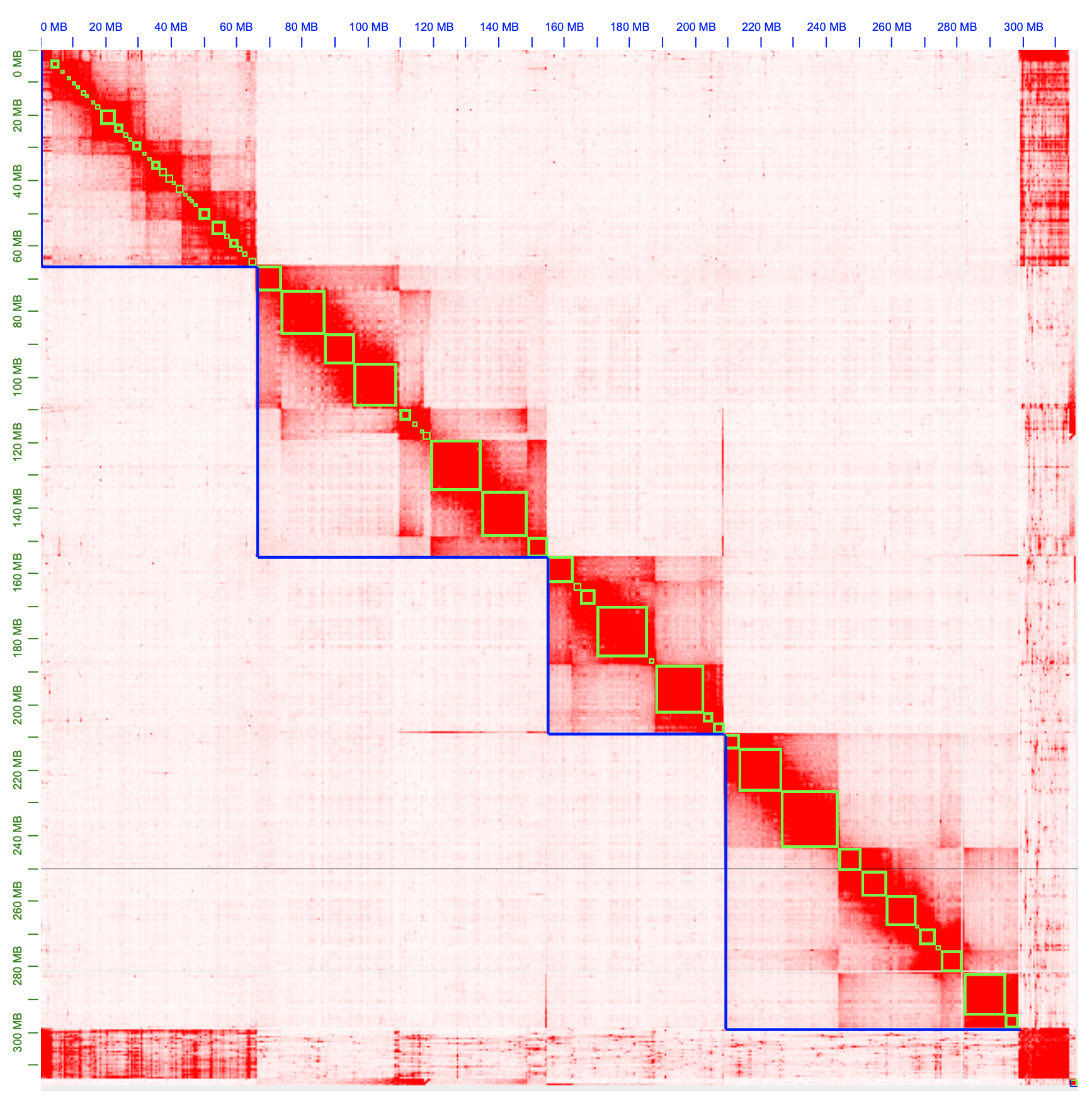


**Figure S7:** Hi-C contact map for the *R. padi* v1 genome assembly. Blue lines show chromosome-scale super scaffolds, green lines show scaffolds from the 10x Genomics lined-read assembly. The x- and y-axis show cumulative length in millions of base pairs (Mb).


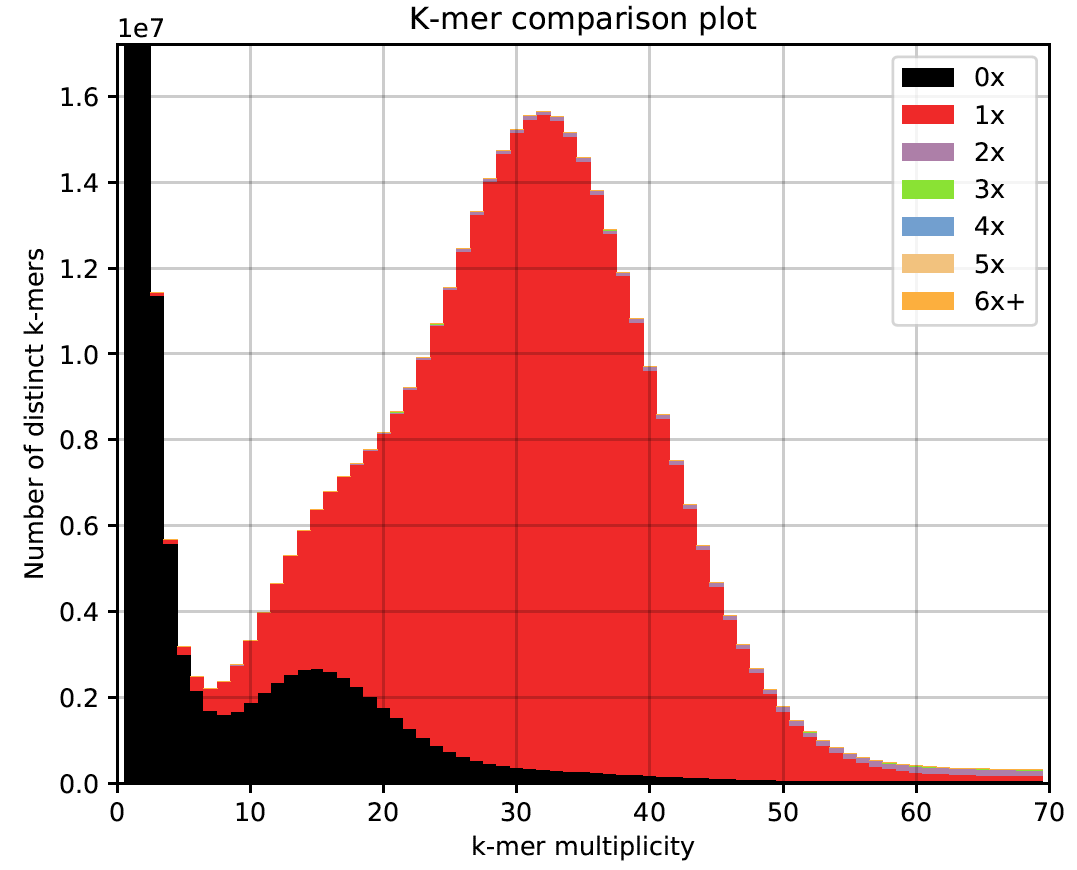


**Figure S8:** KAT k-mer spectra plot comparing *Metopolophium dirhodum* Illumina paired-end (PE) reads to the *M. dirhodum* v1.1 genome assembly. See **Figure S3** legend for detailed description of the plot.


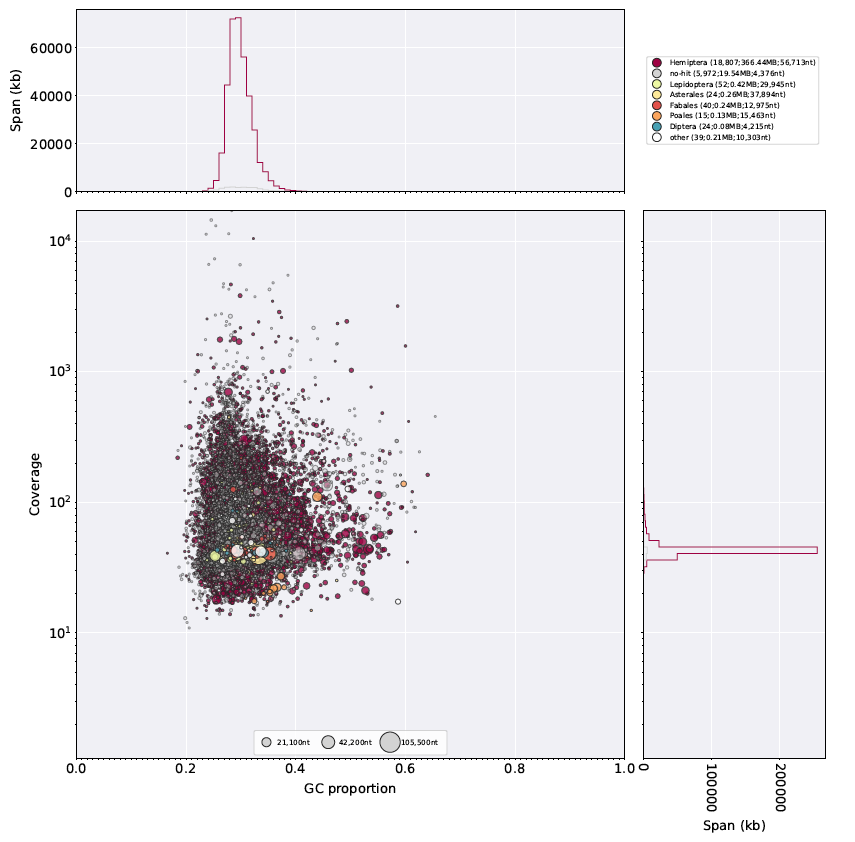
 **Figure S9:** Taxon-annotated GC content-coverage plot of the *Metopolophium dirhodum* v1.1 genome assembly. Scaffold coverage (y-axis) is based on alignment with PCR-free Illumina paired-end reads from the *M. dirhodum* JIC insectary colony. See **Figure S4** legend for detailed description of the plot.


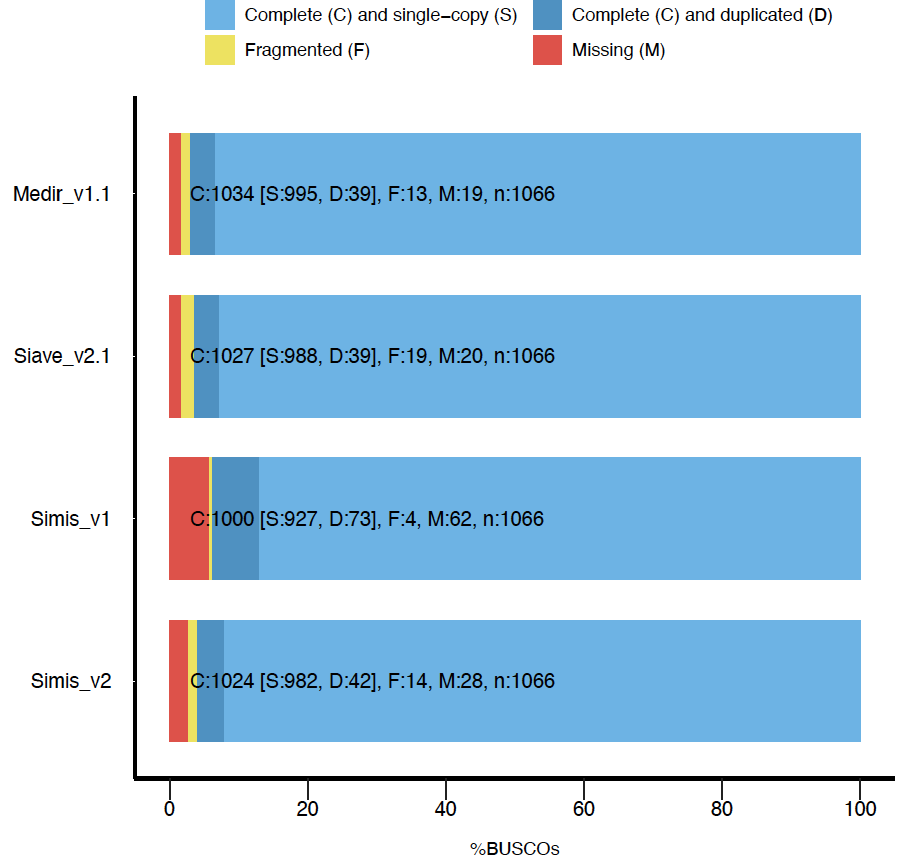


**Figure S10:** BUSCO completeness plot for *Metopolophium dirhodum* v1.1 (Medir_v1.1), *Sitobion avenae* JIC1 v2.1 (Siave_v2.1), *Sitobion miscanthi* v1 (Simis_v1) and *Sitobion miscanthi* v2 (Simis_v2) gene sets. Proteomes from the annotation of each assembly were assessed using BUSCO v3 and the Arthropoda gene set (odb9; n = 1,066). Where multiple transcripts were annotated for a gene, we used the longest transcript.


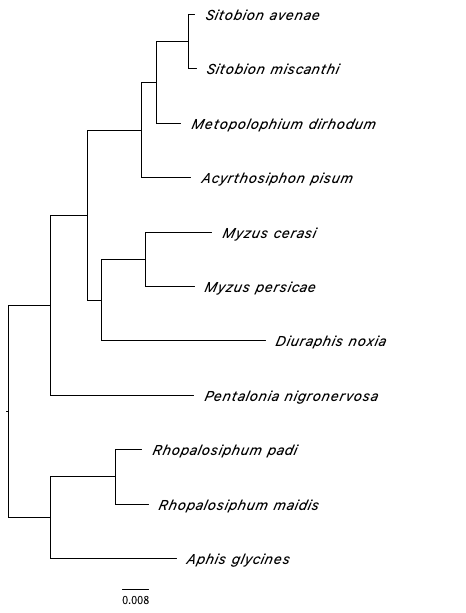


**Figure S11:** Maximum likelihood phylogeny of 11 aphid species with sequenced genomes from the subfamily Aphidinae based on a concatenated alignment of 5,091 conserved one-to-one orthologues. The tree is rooted based on evidence from gene duplications with STRIDE. The basal node corresponds to the split between the tribes Macrosiphini and Aphidini. All nodes received maximal support according to the Shimodaira-Hasegawa test implemented in FastTree with 1,000 resamples. Branch lengths are in amino acid substitutions per site.


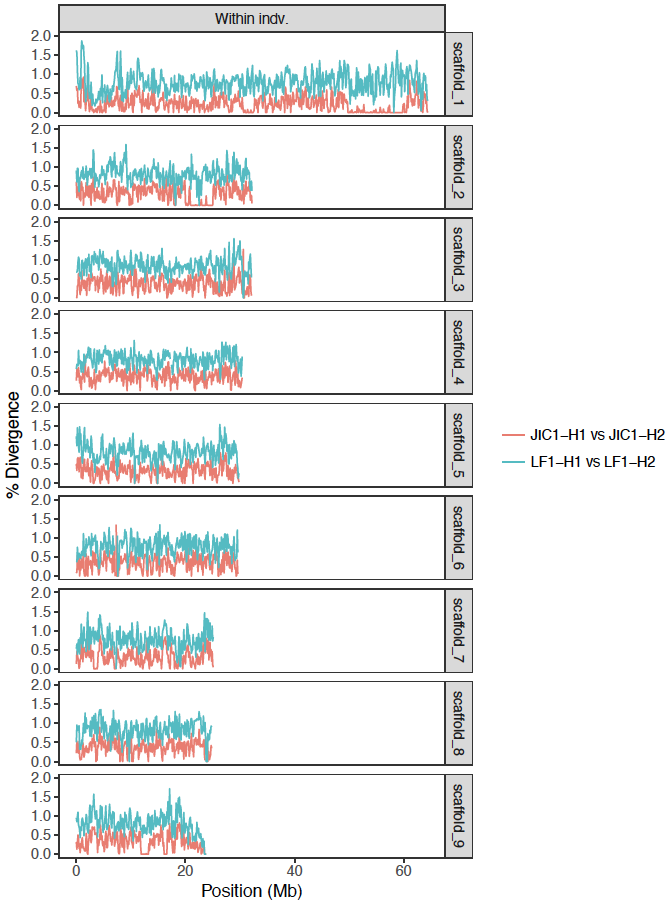


**Figure S12:** JIC1 and LF1 within individual haplotype divergence (all chromosomes).


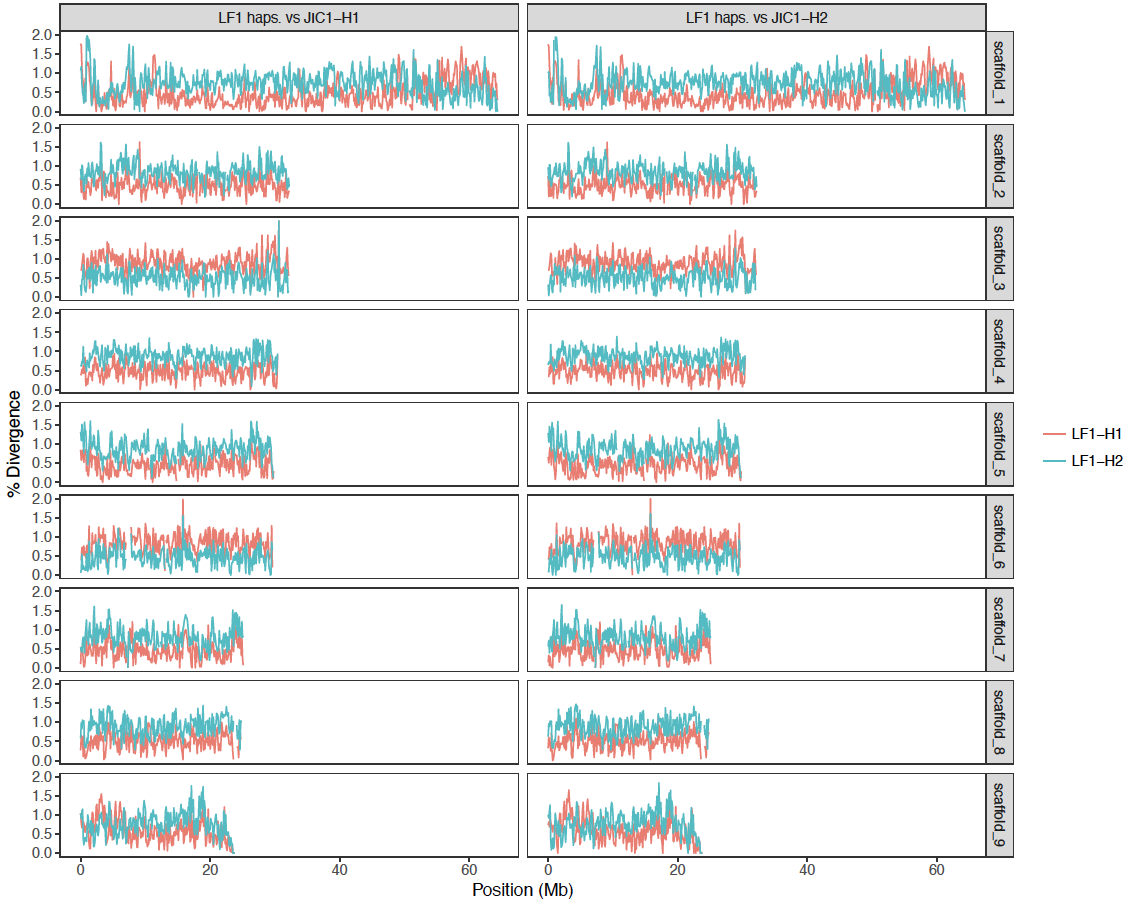


**Figure S13:** JIC1 and LF1 between individual haplotype divergence (all chromosomes).


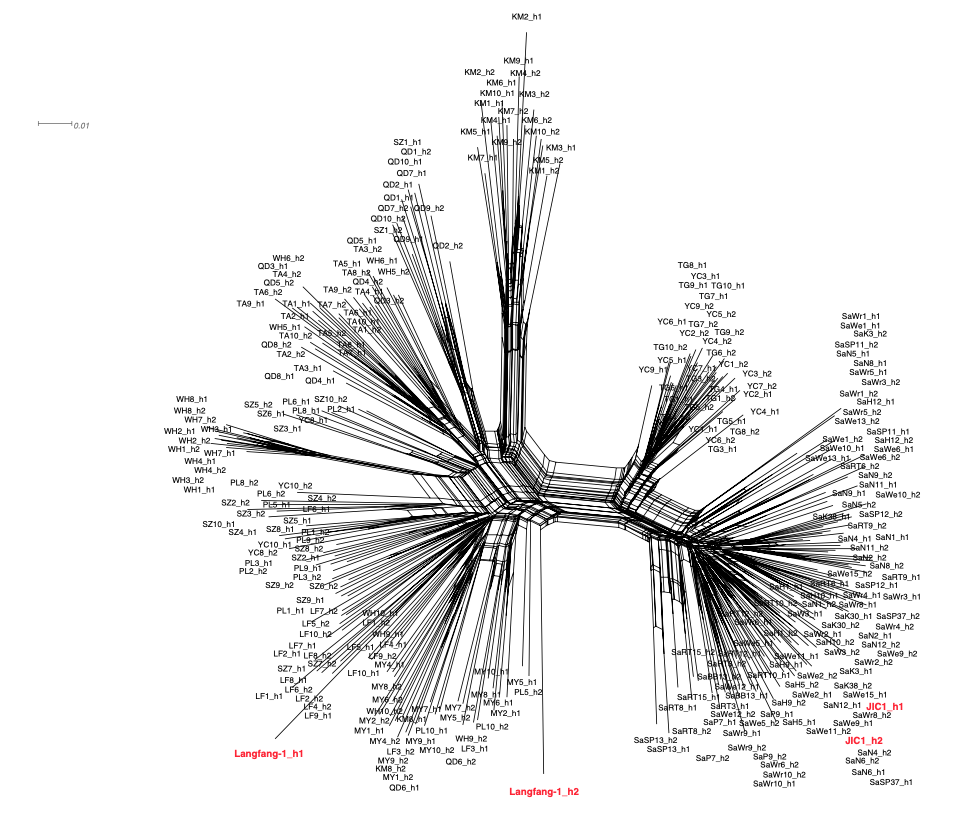


**Figure S14:** SplitsTree network of phased haplotypes for *S. miscanthi* and *S. avaenae* GBS samples and the Langfang-1 and JIC1 whole genome samples (red tip labels). The networked was constructed using SNPs from the “shared SNP set” (see main text) located on the *S. aveane* chromosome X (scaffold_1).


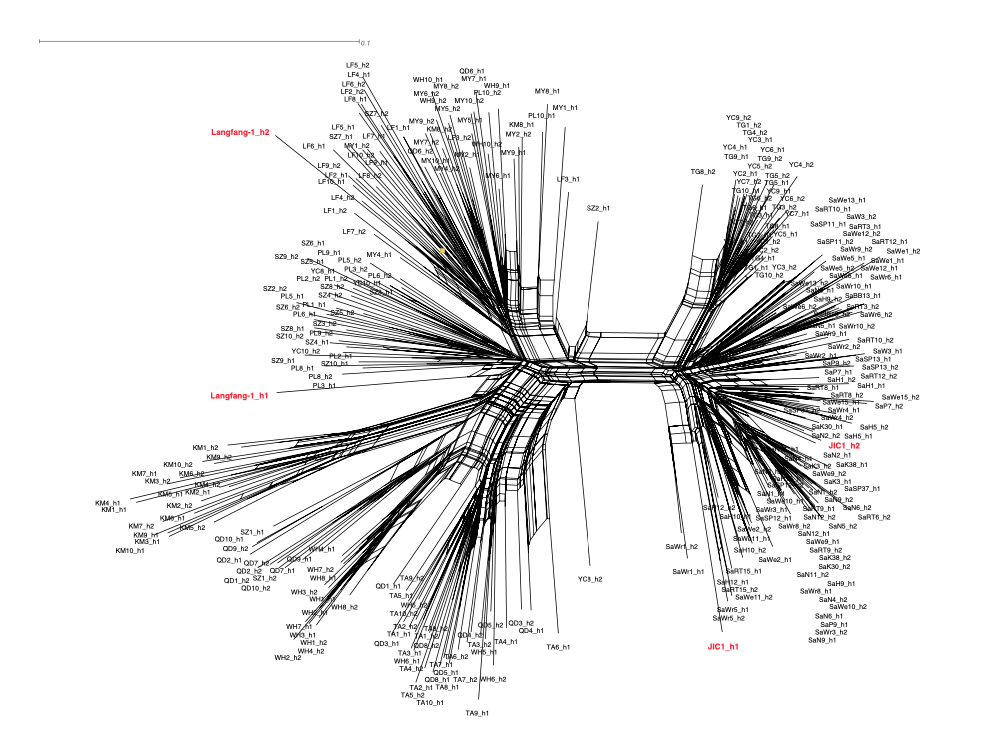


**Figure S15:** As for **Figure S14** but for located on *S. aveane* chromosome 3 (scaffold_3).


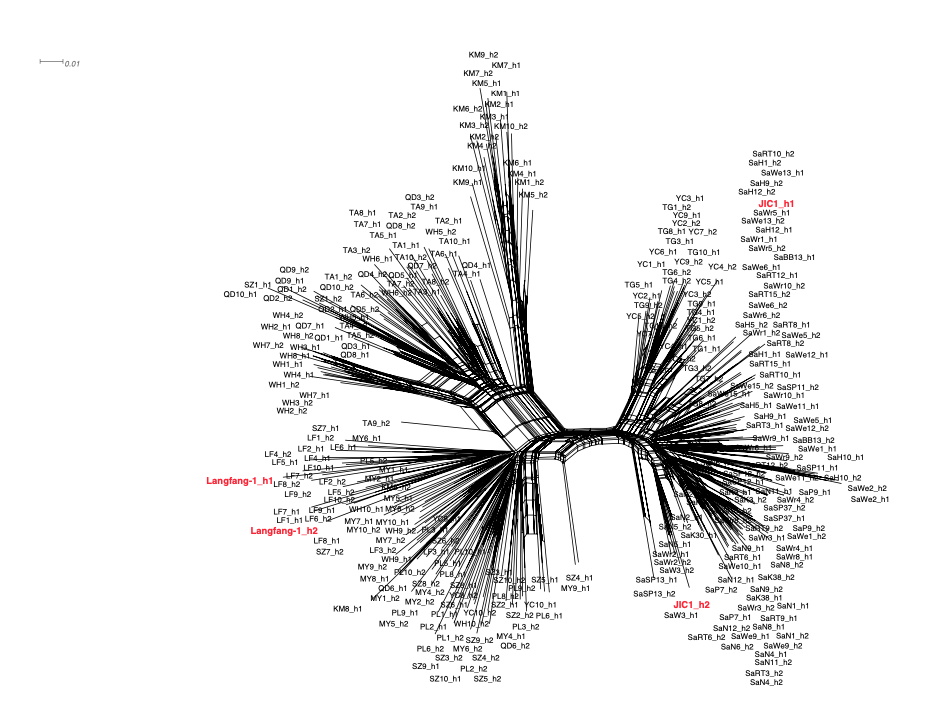


**Figure S16:** As for **Figure S14** but for located on *S. aveane* chromosome 4 (scaffold_4).


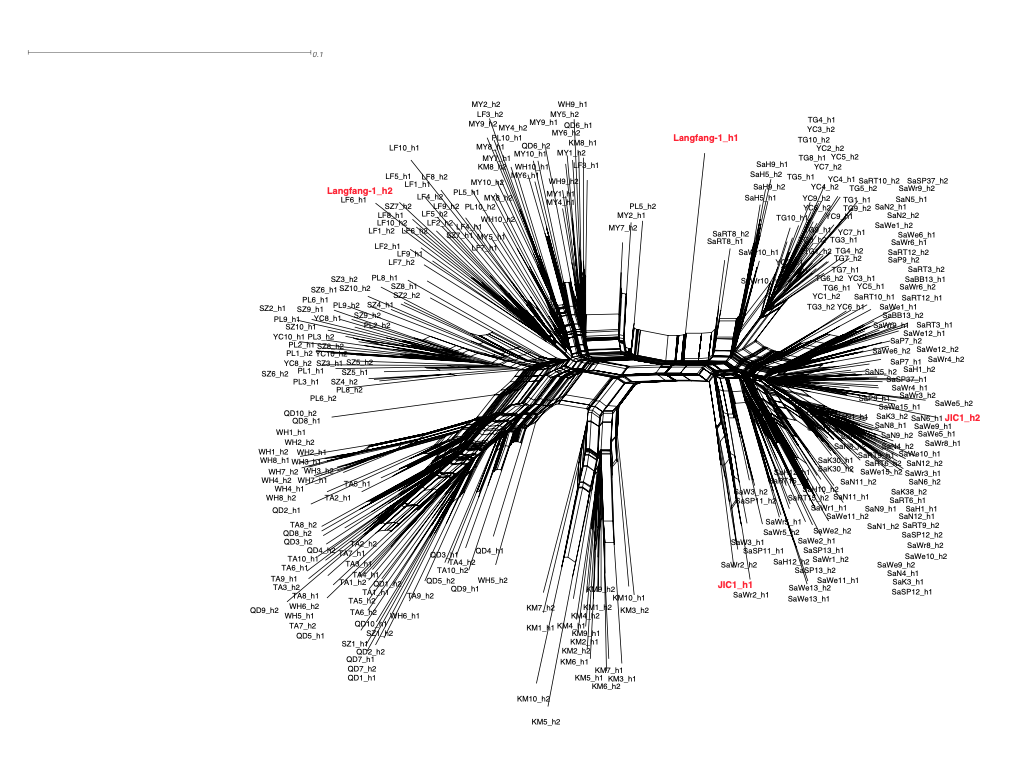


**Figure S17:** As for **Figure S14** but for located on *S. aveane* chromosome 5 (scaffold_5).


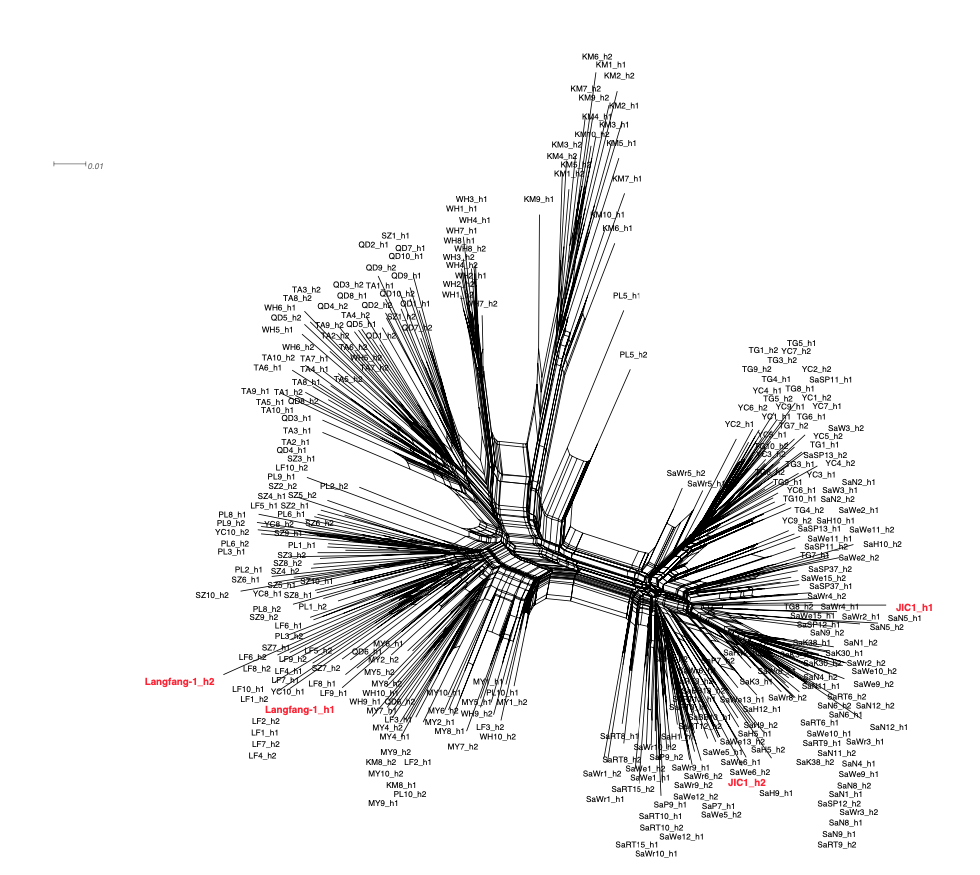


**Figure S18:** As for **Figure S14** but for located on *S. aveane* chromosome 6 (scaffold_6).


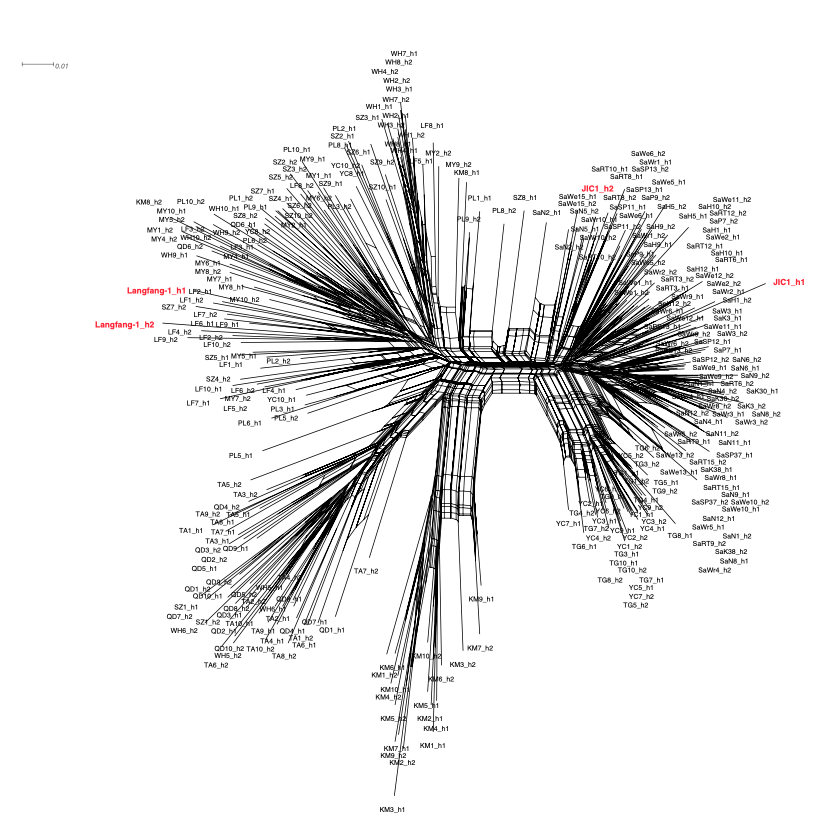


**Figure S19:** As for **Figure S14** but for located on *S. aveane* chromosome 7 (scaffold_7).


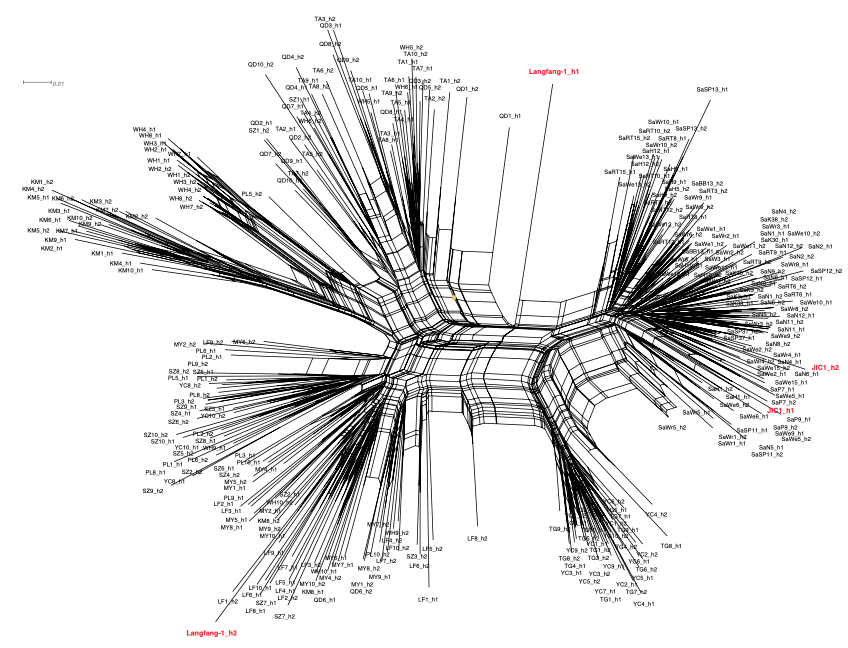


**Figure S20:** As for **Figure S14** but for located on *S. aveane* chromosome 8 (scaffold_8).


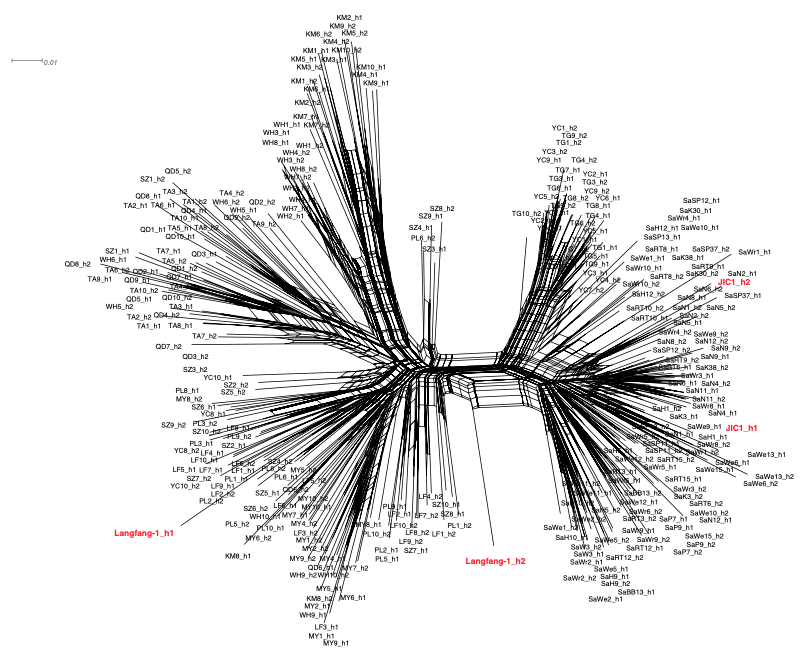


**Figure S21:** As for **Figure S14** but for located on *S. aveane* chromosome 9 (scaffold_9).


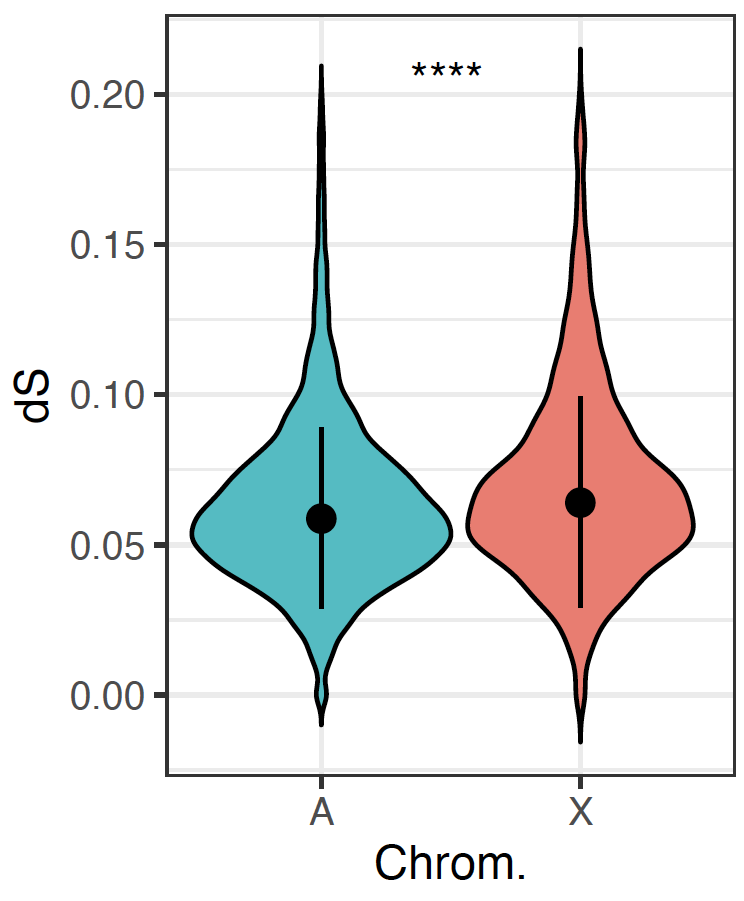


**Figure S22:** *Violin plots* of synonymous site divergence (dS) between *S. miscanthi* and *M. dirhodum* one-to-one orthologs located on *S. miscanthi* autosomes (A; n = 8,462) and the *S. miscanthi* X chromosome (X; n = 1,871). Dot and whiskers show the median and interquartile range, respectively. X chromosome genes have significantly higher dS than autosomal genes (Wilcoxon rank sum test, p = < 2.2x10^-16^, W = 8,879,400). Ortholog pairs with extreme dS values (dS >= 0.2) were omitted.


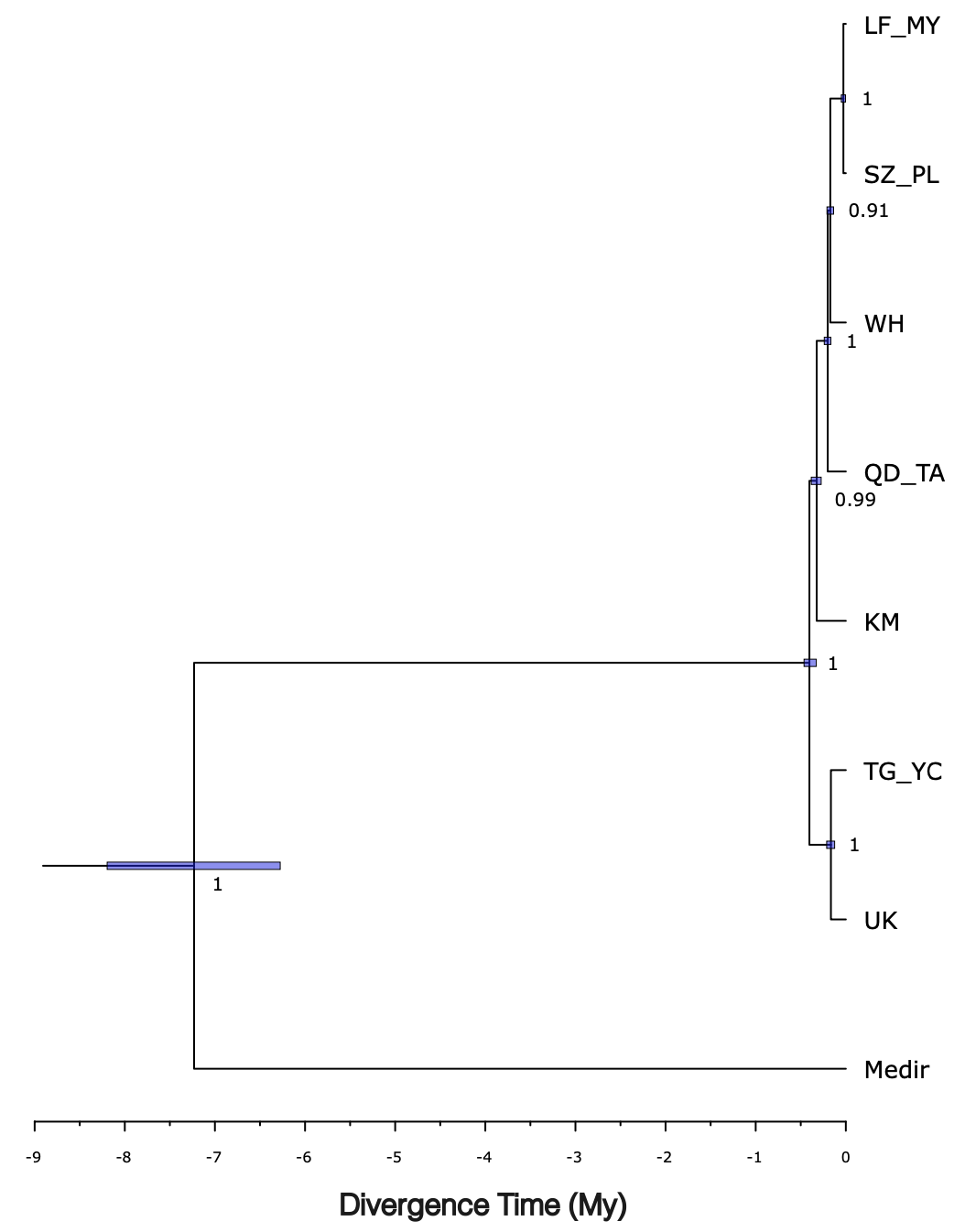


**Figure S23:** SNAPP maximum-clade-credibility time calibrated phylogeny of *Sitobion* lineages rooted with *M.* *dirhodum* (Medir). Lineages are named according to **Figure 5** (*main text*). Bars at nodes indicate 95% highest posterior densities of date estimates. Numbers at nodes show posterior probabilities. My = millions of years ago.

**
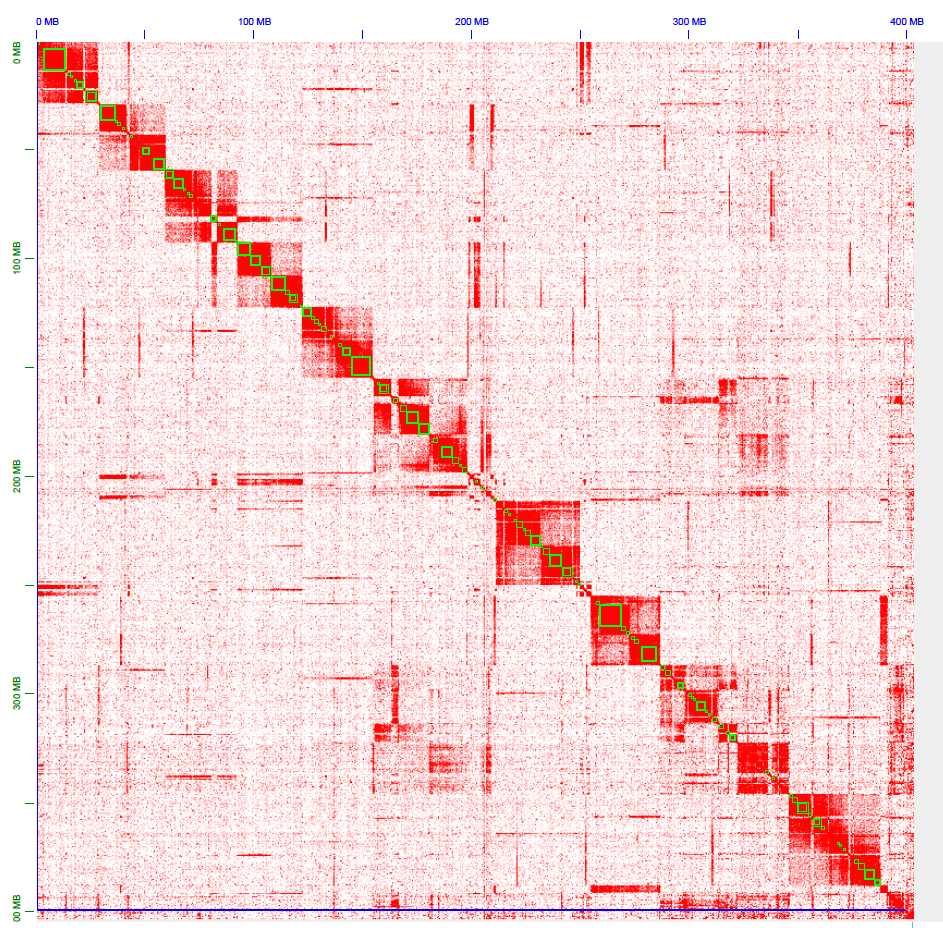
**

**Figure S24:** Hi-C contact map for the draft *S. miscanthi* genome assembly after the initial round of scaffolding carried out by the 3D-DNA pipeline. The blue line shows the single super-scaffold assembled by 3D-DNA that is subsequently broken down into putative chromosome-scale scaffolds in later rounds of the pipeline. The green lines show assembly contigs. Due to poor scaffolding performance likely caused by low resolution of the Hi-C library, we manually edited the assembly in JBAT after this stage of the 3D-DNA pipeline to generate chromosome-scale scaffolds (see *main text* **Figure 1b**).

**
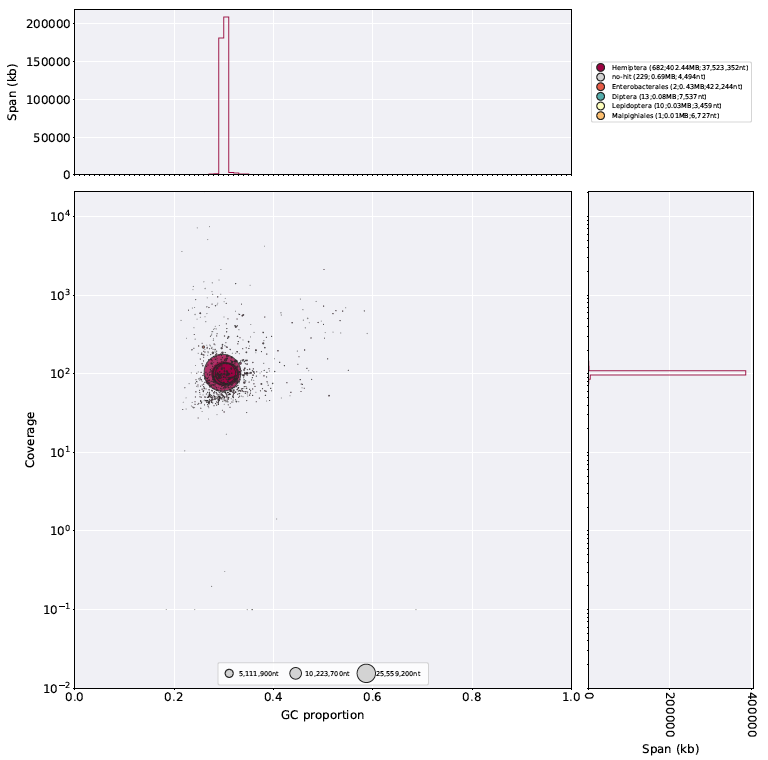
 Figure S25:** Taxon-annotated GC content-coverage plot of the *Sitobion miscanthi* draft genome assembly after Hi-C scaffolding but before removal of symbionts and other contamination. Scaffold coverage (y-axis) is based on alignment of alignment Illumina paired-end reads from the *S. miscanthi* Langfang-1 colony (from Jiang *et al.* 2019). Taxonomy is annotated at the order level. See **Figure S4** legend for detailed description of the plot.


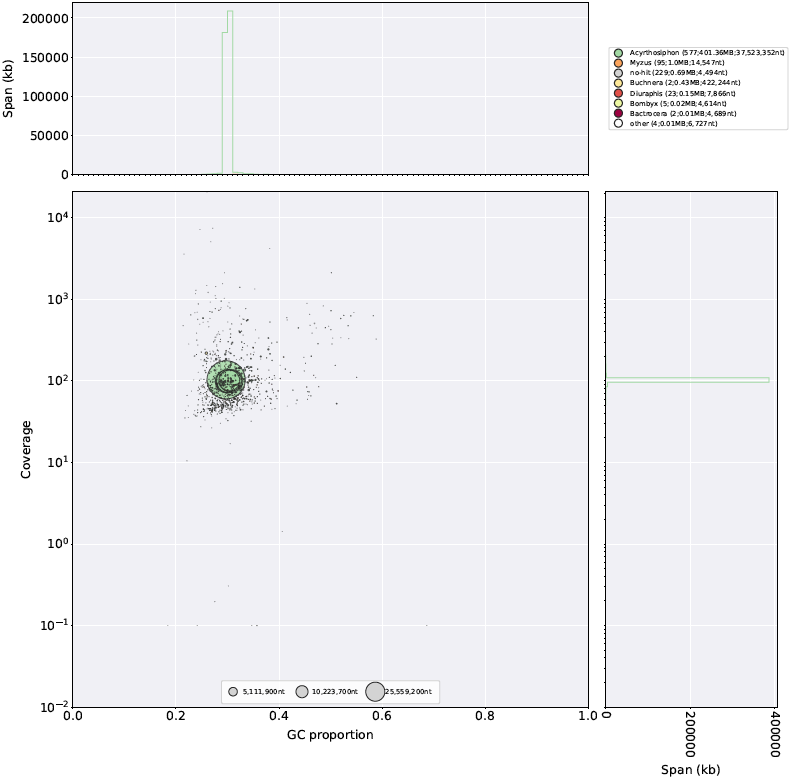


**Figure S26:** As for **Figure S25** but with taxonomy annotated at the genus level.


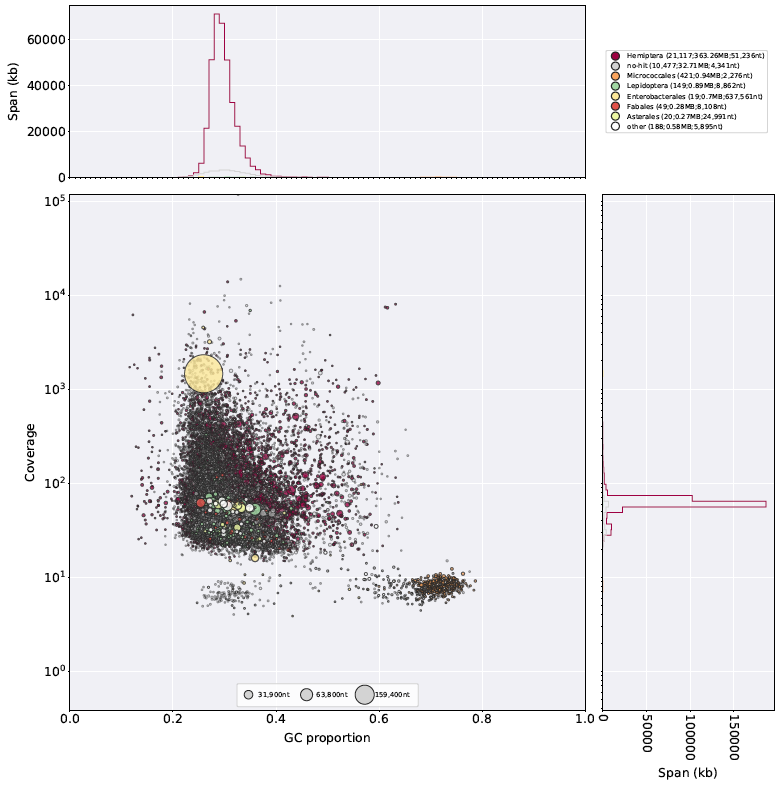


**Figure S27:** Taxon-annotated GC content-coverage plot of the *S. avenae* Discovar *de novo* draft genome assembly after initial deduplication but before removal of symbionts and other contamination. Scaffold coverage (y-axis) is based on alignment of PCR-free Illumina paired-end reads used for the assembly. Taxonomy is annotated at the order level. See **Figure S4** legend for detailed description of the plot.

**
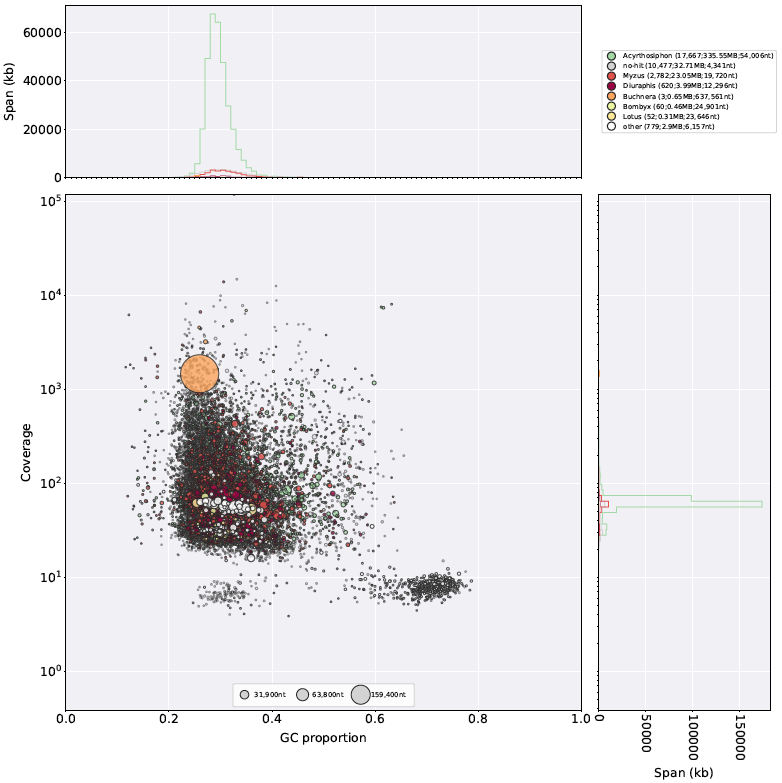
**

**Figure S28:** As for **Figure S27** but with taxonomy annotated at the genus level.


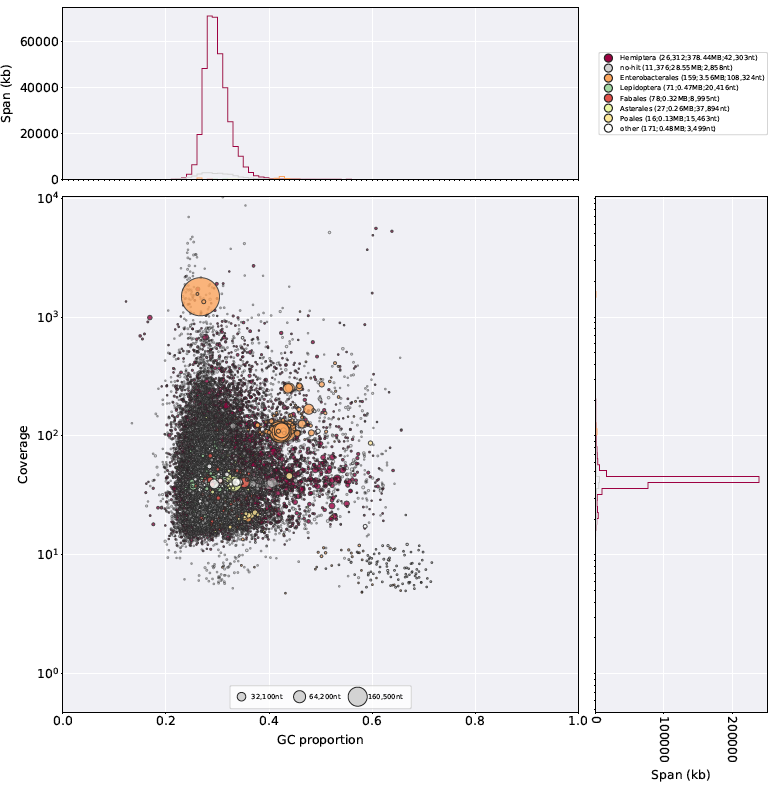


**Figure S29:** Taxon-annotated GC content-coverage plot of the *M. dirhodum* Discovar *de novo* draft genome assembly after initial deduplication but before removal of symbionts and other contamination. Scaffold coverage (y-axis) is based on alignment of PCR-free Illumina paired-end reads used for the assembly. Taxonomy is annotated at the order level. See **Figure S4** legend for detailed description of the plot.


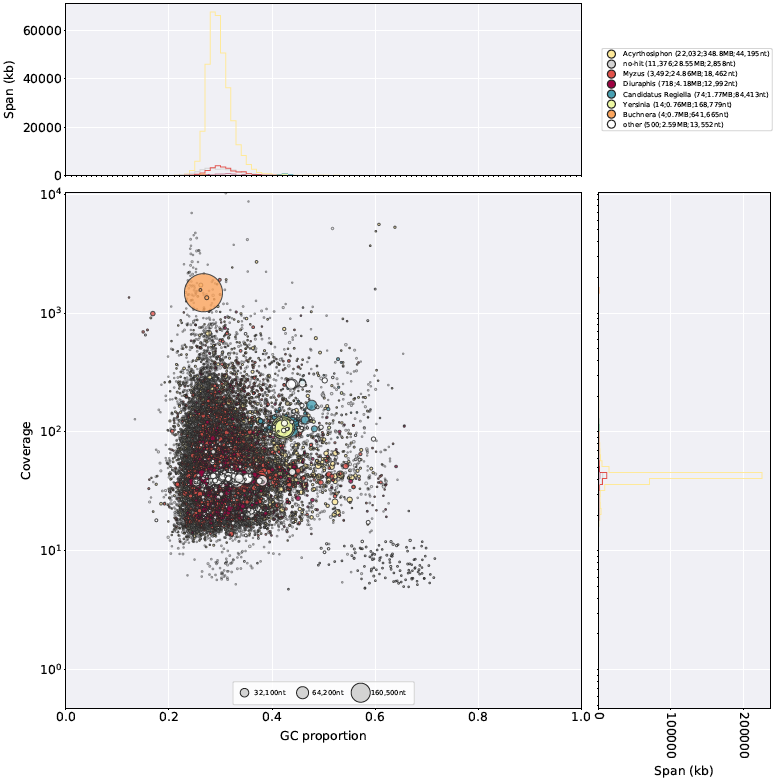


**Figure S30:** As for **Figure S29** but with taxonomy annotated at the genus level.

**
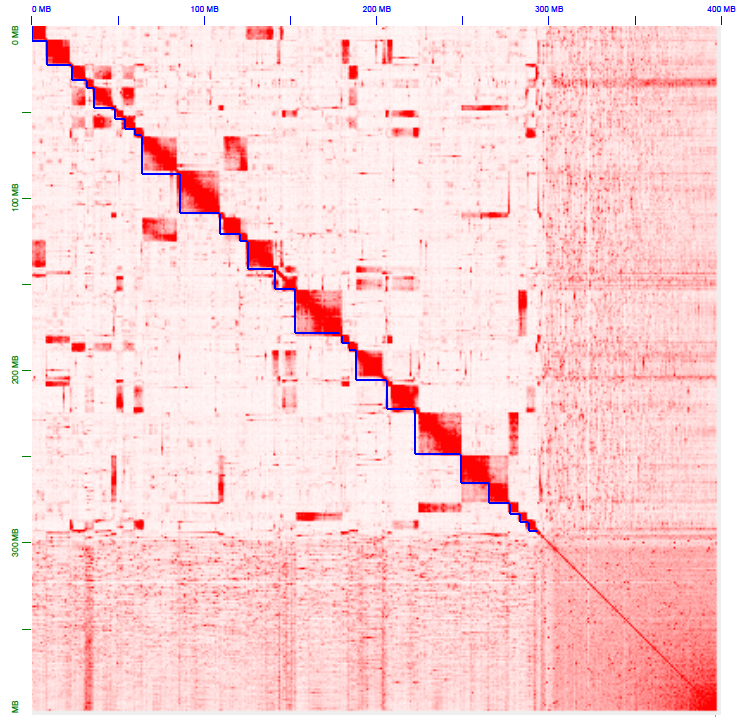
**

**Figure S31:** Hi-C contact map for the 3D-DNA scaffolded draft *S. avenae* assembly before manual review with JBAT. Blue lines indicate super scaffolds.


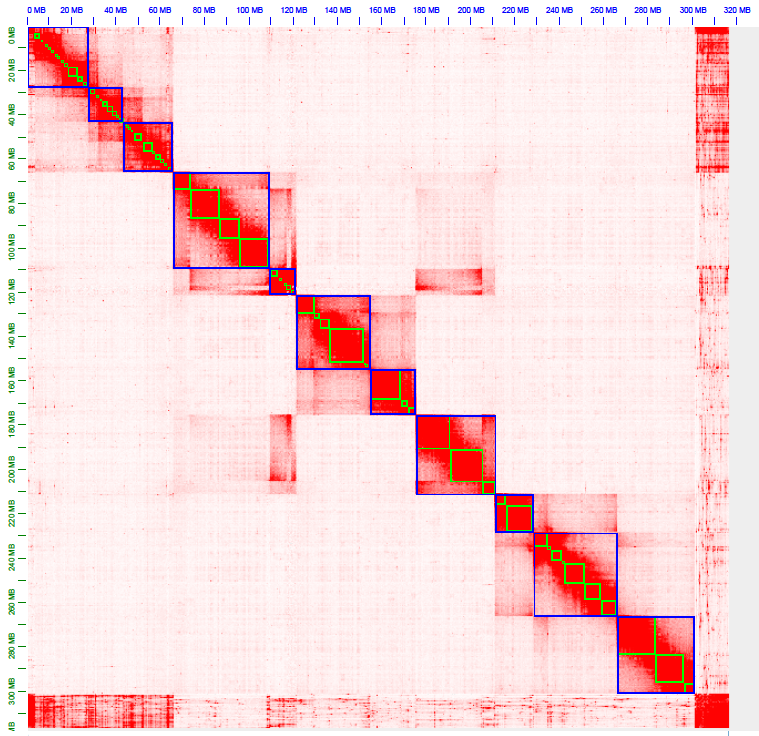


**Figure S32:** Hi-C contact map for the 3D-DNA scaffolded draft *R. padi* assembly before manual review with JBAT. Blue lines indicate super scaffolds, green lines indicate scaffolds in the input draft assembly (Supernova + scaff10x + tigmint).


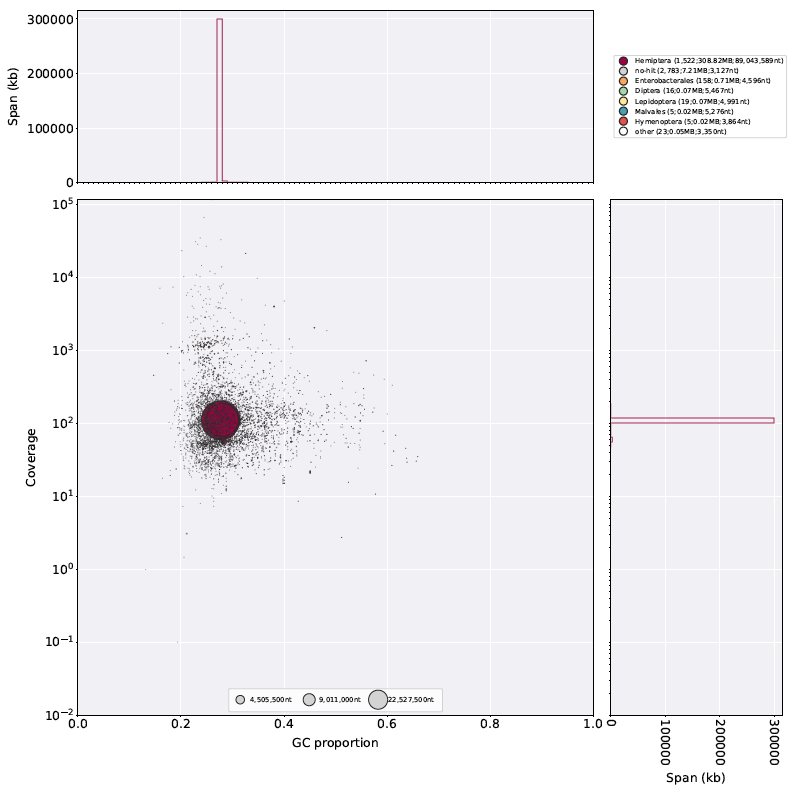
 **Figure S33:** Taxon-annotated GC content-coverage plot of the *R. padi* Discovar *de novo* draft genome assembly after after Hi-C scaffolding but before removal of symbionts and other contamination. Scaffold coverage (y-axis) is based on alignment of 10x genomics linked-reads used for the *de novo* assembly. Taxonomy is annotated at the order level. See **Figure S4** legend for detailed description of the plot.


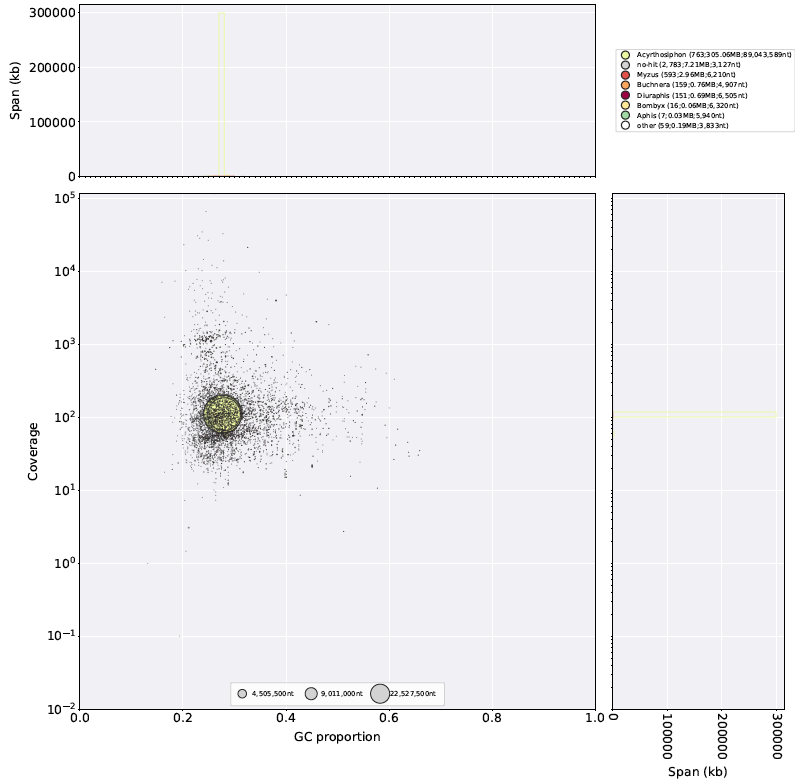


**Figure S34:** As for **Figure S33** but with taxonomy annotated at the genus level.


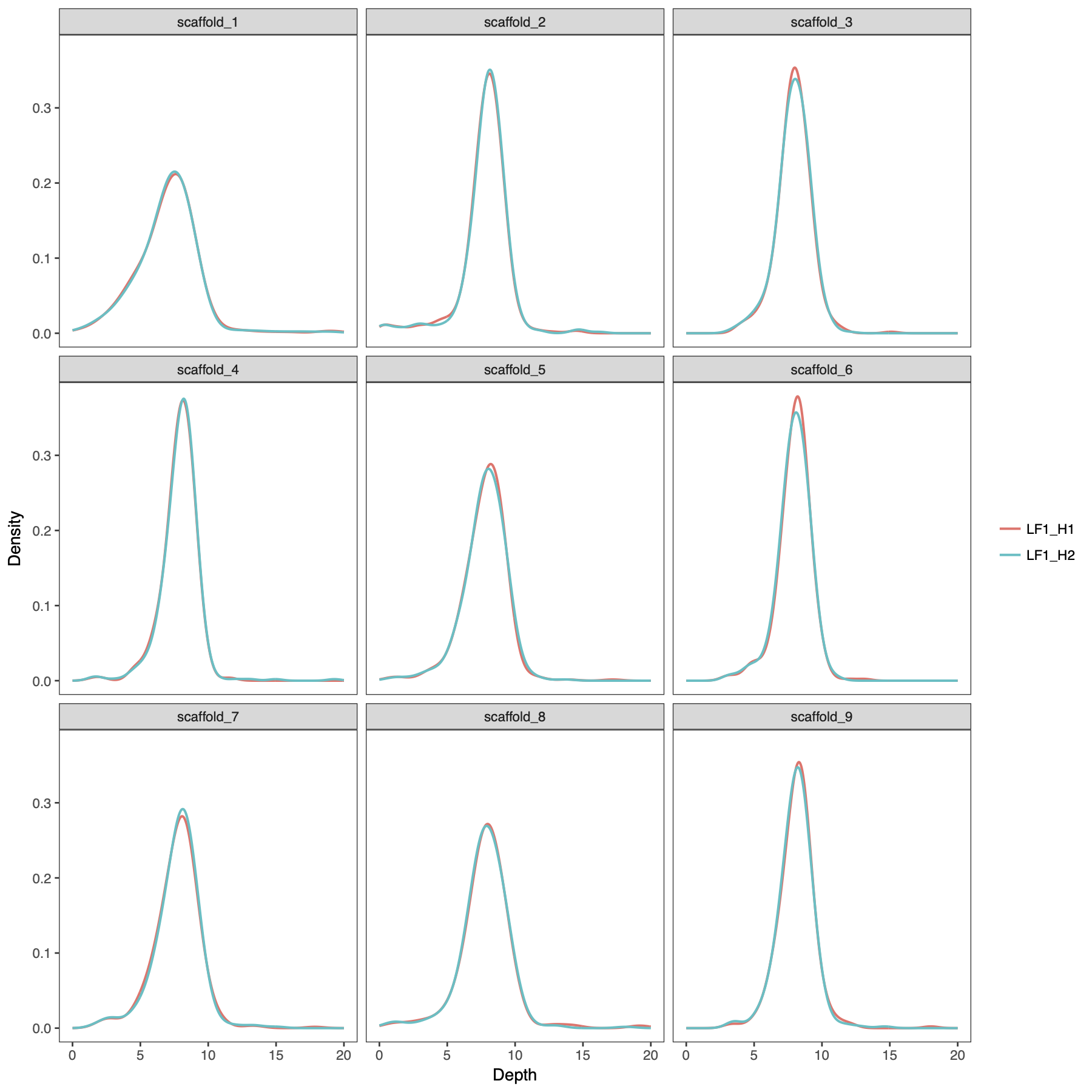


**Figure S35:** *Density plots* of per haplotype, per chromosome sequencing depth (estimated in 100 Kb fixed windows) for the Hapcut2 phased assembly of *S. miscanthi* Langfang-1. Sequencing depth was estimated based on mapping sub-sampled Langfang-1 Illumina short-reads to a merged assembly of Langfang-1 haplotype 1 and haplotype 2.

**
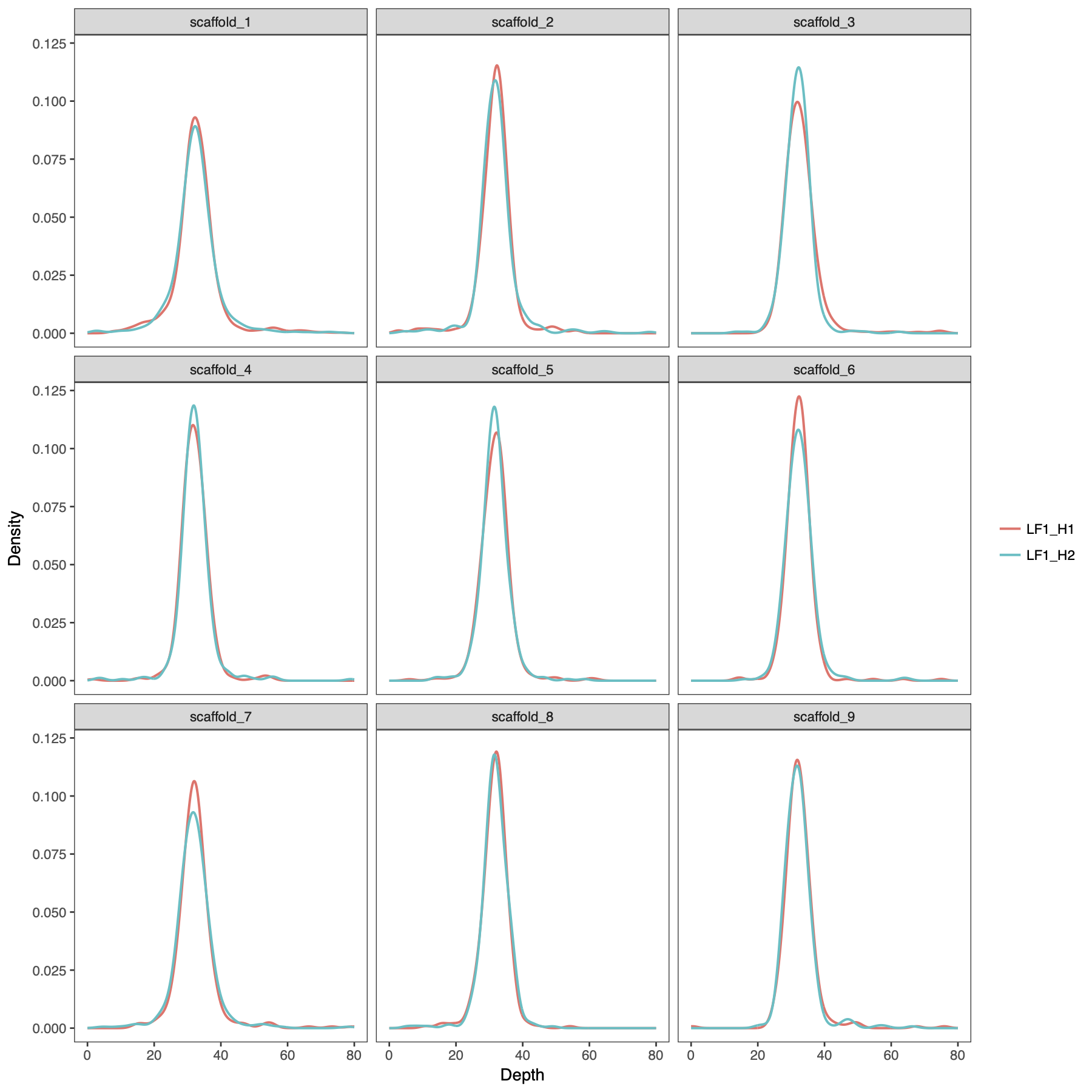
**

**Figure S36:** As for **Figure S35**, but with read depth estimated based on mapping of Langfang-1 PacBio long-reads.
